# Implantable defibrillator therapy and mortality in patients with non-ischaemic dilated cardiomyopathy: An updated meta-analysis and effect on Dutch clinical practice by the Task Force of the Dutch Society of Cardiology

**DOI:** 10.1007/s12471-022-01718-3

**Published:** 2022-09-06

**Authors:** D. A. Theuns, T. E. Verstraelen, A. C. J. van der Lingen, P. P. Delnoy, C. P. Allaart, L. van Erven, A. H. Maass, K. Vernooy, A. A. M. Wilde, E. Boersma, J. G. Meeder

**Affiliations:** 1 Department of Cardiology, Erasmus MC, Rotterdam, The Netherlands; 2 Amsterdam UMC, AMC, Amsterdam, The Netherlands; 3 Amsterdam UMC, Vrije Universiteit, Amsterdam, The Netherlands; 4 Isala klinieken, Zwolle, The Netherlands; 5 LUMC, Leiden, The Netherlands; 6 UMCG, Groningen, The Netherlands; 7 Maastricht University Medical Center (MUMC+), Maastricht, The Netherlands; 8 Cardiovascular Research Institute Maastricht (CARIM), Maastricht, The Netherlands; 9 VieCuri, Venlo, The Netherlands

**Keywords:** Implantable cardioverter-defibrillator, Cardiac resynchronisation therapy, Mortality, Non-ischaemic cardiomyopathy, Meta-analysis, Systematic review

## Abstract

**Background:**

Primary prophylactic implantable cardioverter-defibrillators (ICDs) in patients with non-ischaemic cardiomyopathy (NICMP) remains controversial. This study sought to assess the benefit of ICD therapy with or without cardiac resynchronisation therapy (CRT) in patients with NICMP. In addition, data were compared with real-world clinical data to perform a risk/benefit analysis.

**Methods:**

Relevant randomised clinical trials (RCTs) published in meta-analyses since DANISH, and in PubMed, EMBASE and Cochrane databases from 2016 to 2020 were identified. The benefit of ICD therapy stratified by CRT use was assessed using random effects meta-analysis techniques.

**Results:**

Six RCTs were included in the meta-analysis. Among patients without CRT, ICD use was associated with a 24% reduction in mortality (hazard ratio [HR]: 0.76; 95% confidence interval [CI]: 0.62–0.93; *P* = 0.008). In contrast, among patients with CRT, a CRT-defibrillator was not associated with reduced mortality (HR: 0.74, 95% CI 0.47–1.16; *P* = 0.19). For ICD therapy without CRT, absolute risk reduction at 3‑years follow-up was 3.7% yielding a number needed to treat of 27.

**Conclusion:**

ICD use significantly improved survival among patients with NICMP who are not eligible for CRT. Considering CRT, the addition of defibrillator therapy was not significantly associated with mortality benefit compared with CRT pacemaker.

**Supplementary Information:**

The online version of this article (10.1007/s12471-022-01718-3) contains supplementary material, which is available to authorized users.

## Introduction

According to current European and American guidelines, implantable cardioverter-defibrillator (ICD) therapy is recommended for patients with either ischaemic or non-ischaemic cardiomyopathy (NICMP), reduced left ventricular ejection fraction (LVEF) of ≤ 35%, and New York Heart Association (NYHA) functional class II or III symptoms on optimal medical therapy [1, 2]. The evidence for ICD benefit in patients with ischaemic cardiomyopathy is robust as proven in randomised clinical trials (RCTs) [3, 4]. However, the benefit of ICD implantation in patients with NICMP remains under debate. An early meta-analysis by Desai et al. demonstrated that ICD therapy in patients with NICMP was effective in reducing all-cause mortality; risk ratio (RR) 0.69, 95% CI 0.55–0.87 [5]. In the Sudden Cardiac Death in Heart Failure Trial (SCD-HeFT), the ICD had a non-significant protective effect for mortality compared with placebo among patients with NICMP; hazard ratio (HR) 0.73, 95% CI 0.50–1.07 [3]. The results of the Danish Study to Assess the Efficacy of ICDs in Patients with Non-ischemic Systolic Heart Failure on Mortality (DANISH) showed that ICD therapy was associated with a reduction in SCD but not in all-cause mortality [6]. However, 58% of the patients in the DANISH trial received cardiac resynchronisation therapy (CRT), which might have confounded the results by improving LVEF in some patients. In selected patients, CRT may reduce both sudden and non-sudden death when compared with medical therapy (MT). In general, patients who qualify for CRT have a higher co-morbidity burden and more advanced heart failure compared with those who qualify for ICD therapy. It is imperative to understand the value of ICD in NICMP patients with and without CRT. Previous meta-analyses of ICD trials showed conflicting results, as data of ICD and CRT‑D were combined and analyses were mixed CRT versus MT and CRT‑D versus CRT‑P [7–24]. Therefore, analyses to assess the benefit of ICD therapy should be stratified according to CRT use. We performed a systematic review and meta-analysis of RCTs to assess the benefit of ICD therapy on all-cause mortality in patients with NICMP, either with or without CRT. Relative risk estimates were then applied to real-world data from Dutch clinical practices in order to gain insight in the absolute risk reduction (ARR) and the number needed to treat (NNT) to prevent one death during 3‑year follow-up.

## Methods

### Search strategy

We performed a modified literature search to identify all RCTs that were included in previous meta-analyses, including DANISH. An overview of published meta-analyses after DANISH is presented in Supplementary Table S1. In addition, we searched the public domain databases PubMed, EMBASE, and the Cochrane Central Register of Clinical Trials to identify RCTs assessing benefit of ICD for primary prevention of SCD in patients with NICMP between September 1, 2016, through December 31, 2020. We used the terms ‘implantable cardioverter-defibrillator, dilated cardiomyopathy, non-ischemic cardiomyopathy, and primary prevention’. The reference list of identified articles was also reviewed. Studies with less than 100 participants were excluded.

### Data extraction and quality assessment

Data from eligible RCTs were independently abstracted by two reviewers (AvdL, DAT) using a structured form. Data included eligibility criteria, period of enrolment, type of experiment and comparison group (ICD and/or CRT‑D versus active control, placebo or MT), duration of follow-up, proportion of crossover, patient demographics and clinical characteristics, and outcome data, including all-cause mortality (primary) and SCD (secondary).

Two reviewers (AvdL, TEV) independently assessed risk of bias using the CASP Randomised Controlled Trial Standard Checklist. This checklist includes design of the RCT (study population, comparator and measured outcomes), allocation concealment, intention-to-treat analysis, early study termination, blinding, equal intervention during follow-up, adequacy of statistical analysis and absence of selective reporting. Disagreements were discussed and a final decision was reached by consultation with a third reviewer (DAT) if disagreements were not resolved.

### Data analysis

Summary statistics from the individual trials were used as patient-level data were not available for all studies. Descriptive analyses were conducted using weighted means and standard deviations for continuous variables and weighted frequencies for categorical variables.

We performed meta-analyses comparing 1) ICD-only with MT, 2) CRT‑D with MT, and 3) CRT‑D with CRT‑P, while applying the intention-to-treat principle. For all-cause mortality, we calculated the pooled estimate of HR by using the reported HRs with corresponding 95% confidence intervals (CIs). Regarding SCD, we calculated odds ratios (ORs) by the raw data provided in these studies, since HRs were not reported uniformly among studies. We applied the random-effects model according to DerSimonian and Laird [25]. Evidence of statistical heterogeneity between studies was checked and quantified by the inconsistency index (*I*^2^) statistic. *I*^2^ values less than 25% and *I*^2^ greater than 75%, were considered as low and high heterogeneity respectively.

We performed sensitivity analyses to assess the contribution of individual trials to the pooled estimate by recalculating the pooled estimate after exclusion of the corresponding trial(s). First, AMIOVIRT was excluded, since it is the only trial that also included hypertrophic cardiomyopathy, sarcoidosis and myocarditis, while other trials excluded those patients. Second, CAT and AMIOVIRT were excluded, as both trials were halted due to statistical futility. We performed further sensitivity analyses to evaluate the effect of length of follow-up by comparing trials with < 3 years of follow-up versus those with ≥ 3 years of follow-up. In addition, we performed sensitivity analysis to test the effect of amiodaron by comparing pooled analyses of trials with and without amiodaron. Potential publication bias was assessed by visually examining the funnel plot. Pooled data analysis was performed with Cochrane Review Manager (release 5.4, the Cochrane Collaboration, Copenhagen, Denmark). For all analyses, a *P*-value ≤ 0.05 was considered statistically significant.

We then combined the results, estimates of relative mortality reduction, of the current meta-analysis with the data from the Dutch outcome in ICD therapy (DO-IT) registry to obtain estimates of absolute mortality reduction (ARR) and the number needed to treat (NNT) to prevent one death that are relevant for the Dutch outcome. The DO-IT registry is a recent primary prevention ICD study which recruited 1,640 patients reflective of current practice, to establish current baseline mortality risk of general NICMP patients [26]. Using the 3‑year follow-up data of the DO-IT registry, the cumulative incidence of mortality was calculated for NICMP patients with ICD-only therapy. Subsequently, we used the pooled HR of ICD-only therapy versus MT that was obtained in the current meta-analysis to estimate the cumulative mortality had the DO-IT patients received MT.

## Results

### Study selection

The initial database search yielded 2,884 articles and after removing duplicates, 2,563 potential articles were further screened (Supplementary Fig. S1). After screening of titles and abstract, 34 articles were eligible for full text screening. A total of 7 RCTs were identified in previous meta-analyses, from which we included 6 for the current meta-analysis. One RCT, Pro-ICD, was excluded as the study only enrolled 19 patients [27]. In addition, 2 non-randomised clinical trials were identified and used to assess the benefit of ICD therapy in current real-world clinical practice; the prospective, controlled study EUropean Comparative Effectiveness Research to Assess the Use of Primary ProphylacTic Implantable Cardioverter-Defibrillators (EU-CERT-ICD) and the Swedish Heart Failure Registry (SwedeHF) [28, 29].

### Study characteristics

A total of 6 RCTs were included in the meta-analysis (Tab. 1): the cardiomyopathy trial (CAT), the amiodarone versus implantable defibrillator trial (AMIOVIRT), defibrillators in non-ischemic cardiomyopathy treatment evaluation (DEFINITE), comparison of medical therapy, pacing, and defibrillation in heart failure (COMPANION), SCD-HeFT, and DANISH. Four RCTs exclusively enrolled patients with NICMP and 2 trials (COMPANION and SCD-HeFT) also enrolled patients with ischaemic cardiomyopathy. Two trials had 3 comparison groups each, SCD-HeFT compared ICD versus placebo versus amiodarone, whereas COMPANION compared CRT‑D and CRT‑P versus MT. Considering CRT, DANISH compared ICD (with or without CRT) with MT (with or without CRT). For the current analysis, patients with ischaemic cardiomyopathy from COMPANION and SCD-HeFT were excluded.

**Table 1:** Characteristics of the randomised clinical trials

	CAT [30]	AMIOVIRT [31]	DEFINITE [32]	COMPANION [33]	SCD-HeFT [3]	DANISH [6]
Author	Bansch et al.	Strickberger et al.	Kadish et al.	Bristow et al.	Bardy et al.	Kober et al.
Year of publication	2002	2003	2004	2004	2005	2016
Enrolment period	1991–1997	1996–2000	1998–2002	2000–2002	1997–2001	2008–2014
Number of patients	104	103	458	1,520	2,521	1,116
Control group	MT	Amiodarone + MT	MT	MT	Placebo (MT) or amiodarone + MT	MT or MT + CRT‑P
Inclusion criteria	DCM LVEF ≤ 30% NYHA class II–III	DCM LVEF ≤ 35% NYHA class I–III NSVT	niCMP LVEF ≤ 35% NYHA class I–III NSVT or PVC	iCMP and niCMP LVEF ≤ 35% NYHA class III–IV QRS > 120 ms	iCMP and niCMP LVEF ≤ 35% NYHA class II–III	niCMP LVEF ≤ 35% NYHA class II–IV NT-pro BNP > 200
Exclusion criteria regarding CMP	Myocarditis HCM Restrictive CMP		Familial CMP Congenital HD	Infiltrative CMP HCM	Infiltrative CMP HCM Myocarditis Congenital HD	Myocarditis HCM Congenital HD Constrictive pericarditis
Primary endpoint	All-cause mortality	All-cause mortality	All-cause mortality	Composite of all-cause mortality or hospitalisation for any cause	All-cause mortality	All-cause mortality
Follow-up (years)	2.0	2.2	2.4	1.3	3.8	5.6
Crossover	n. r.	Yes	Yes	Yes	Yes	Yes
ITT analysis	Yes	Yes	Yes	Yes	Yes	Yes

*CMP* cardiomyopathy, *DCM* dilated cardiomyopathy, *HCM* hypertrophic cardiomyopathy, *HD* heart disease, *iCMP* ischemic cardiomyopathy, *ITT* intention-to-treat, *LVEF* left ventricular ejection fraction, *MT* medical therapy, *niCMP* non-ischaemic cardiomyopathy, *n.* *r.* not reported, *NYHA* New York Heart Association, *PVC* premature ventricular complex

The primary outcome in all RCTs, except for COMPANION, was all-cause mortality. The COMPANION trial had a combined primary endpoint of all-cause mortality and HF hospitalisation. The total number of patients included in the current meta-analysis is 3,547 patients including the amiodarone arm of SCD-HeFT. Of these patients, 1,200 were treated with CRT; CRT‑D (*n* = 592) and CRT‑P (*n* = 608). Overall, the weighted mean age was 60.4 ± 4.9 years and the majority were male (73%). The weighted mean LVEF was 23.2 ± 1.7% and 63% had NYHA functional class II. Other baseline clinical characteristics are listed in Tab. 2. Considering CRT, mean QRS duration was 160 ms both in COMPANION and DANISH. NYHA III was more prevalent in COMPANION compared with DANISH, 86% versus 46%.

**Table 2:** Baseline characteristics of patients enrolled in the included randomised clinical trials

	CAT		AMIOVIRT		DEFINITE		COMPANION			SCD-HeFT			DANISH	
	MT	ICD	MT	ICD	MT	ICD	MT	CRTP	CRTD	Placebo	Amio	ICD	MT	ICD
Enrolled patients (*n*)	54	50	52	51	229	229	308	617	595	847	845	829	560	556
*Patient characteristics*														
Age, years	52	52	60	58	58	58	68	67	66	60	60	60	63	64
Male	77	86	74	67	70	73	69	67	67	77	76	77	72	73
AF	11	20	n. r.	n. r.	26	23	n. r.	n. r.	n. r.	14	16	17	20	24
niCMP	100	100	100	100	100	100	41	46	45	47	50	48	100	100
Duration of HF, years	2.5	3	1.8	2.2	3.3	2.4	4.9	n. r.	4.4	n. r.	n. r.	n. r.	1.5	1.7
LVEF, %	25	24	23	22	22	21	22	20	22	25	25	24	25	25
*NYHA class*														
– I			13	18	18	25								
– II	64	67	63	64	61	54				70	71	68	54	53
– III	36	33	24	16	21	21	82	87	86	30	29	32	46	47
QRS duration, ms	114	102	n. r.	n. r.	116	115	158	160	160	n. r.	n. r.	n. r.	145	146
CRT	0	0	0	0	0	0	0	100	100	0	0	0	57	58
*Pharmacological therapy*														
Amiodarone	n. r.	n. r.	100	0	7	4	55	n. r.	55	n. r.	100	n. r.	6	6
Betablocker	4	4	50	53	84	86	66	68	68	69	69	69	92	92
ACE/ARB	98	94	81	90	96	97	89	89	90	98	97	94	97	96
MRA	n. r.	n. r.	19	20	n. r.	n. r.	55	53	55	n. r.	n. r.	20	57	59

Continuous data are presented as mean or median and categorical data as percentage

*ACE* angiotensin-converting enzyme, *AF* atrial fibrillation, *ARB* angiotensin receptor blocker, *CRT* cardiac resynchronisation therapy, *CRT‑D* cardiac resynchronisation defibrillator, *CRT‑P* cardiac resynchronisation pacemaker, *HF* heart failure, *ICD* implantable cardioverter-defibrillator, *LVEF* left ventricular ejection fraction, *MT* medical therapy, *niCMP* non-ischaemic cardiomyopathy, *n.* *r.* not reported, *NYHA* New York Heart Association, *OMT* optimal medical therapy

### Quality assessment and publication bias

The method of sequence generation was adequate, and allocation was adequately concealed. Analysis was performed on an intention-to-treat basis and crossovers were reported. Overall, bias was observed in the blinding of participants or failure of binding reporting and selection bias was present among the trials. Funnel plots did not reveal publication bias for comparison of ICD-only versus MT regarding all-cause mortality and SCD. No publication bias was also observed for comparison of CRT‑D versus CRT‑P regarding all-cause mortality.

### All-cause mortality, ICD-only therapy versus medical therapy

**Fig. 1:**
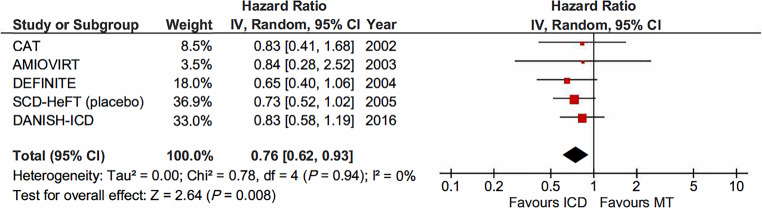
All-cause mortality among patients with non-ischaemic cardiomyopathy randomised to implantable cardioverter-defibrillator (*ICD*) versus medical therapy (*MT*). The hazard ratio (*HR*) of SCD-HeFT represents ICD versus placebo. (*CI* confidence interval, *IV* interval variable, *random* random effect)

All-cause mortality was reported in 5 trials enrolling 1,928 patients with 962 in the ICD group and 966 in the MT group. Pooling data from these 5 trials showed a significant reduction in all-cause mortality with use of an ICD (HR 0.76, 95% CI 0.62–0.93; *I*^2^ = 0%; *P* = 0.008) (Fig. 1).

We performed sensitivity analyses to examine the stability of this finding. First, we excluded AMIOVIRT, which enrolled a heterogeneous cohort of patients with NICMP. Second, we excluded the first two primary prevention trials focussing on NICMP, CAT and AMIOVIRT, which were both halted early due to futility. There was no apparent change in results; (HR 0.76, 95% CI 0.61–0.94) versus (HR 0.75, 95% CI 0.60–0.94). To further examine whether DANISH had an impact on reduction of all-cause mortality, data of the pre-DANISH trials were pooled. All-cause mortality was significantly reduced by ICD-only therapy (HR 0.72, 95% CI 0.56–0.93; *I*^2^ = 0%; *P* = 0.01). Regarding the length of follow-up, pooled analysis of trials with < 3 years follow-up showed a trend towards more benefit of ICD-only therapy (HR 0.68, 95% CI 0.43–1.06 *I*^2^ = 0%; *P* = 0.09) versus those with follow-up ≥ 3 years (HR 0.78, 95% CI 0.62–0.99; *I*^2^ = 0%; *P* = 0.04). However, no difference between groups was found (*P* = 0.58).

Two trials, AMIOVIRT and SCD-HeFT, enrolled patients under amiodarone therapy as control group. Using event data, pooled data-analysis also showed a significant benefit of ICD-only therapy in reducing all-cause mortality (OR 0.73, 95% CI 0.59–0.91; *P* = 0.005; *I*^2^ = 0%). We performed a sensitivity analysis designed to test the effect of amiodarone by comparing the pooled analysis of trials with amiodarone as MT versus those without amiodarone as MT. We found no difference between these groups (*P* = 0.73).

### Sudden cardiac death, ICD-only therapy versus medical therapy

**Fig. 2:**
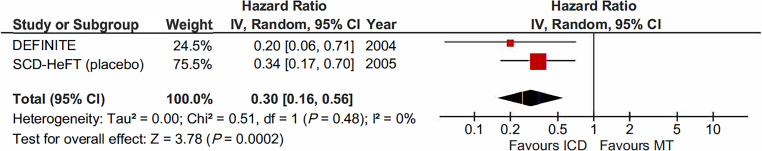
Sudden cardiac death among patients with non-ischaemic cardiomyopathy randomised to implantable cardioverter-defibrillator (*ICD*) versus medical therapy (*MT*). The hazard ratio (*HR*) of SCD-HeFT represents ICD versus placebo. (*CI* confidence interval, *IV* interval variable, *random* random effect)

Two trials, DEFINITE and SCD-HeFT, reported on SCD enrolling 1,250 patients with 627 in the ICD group and 623 in the MT group. The pooled HR for the ICD in reducing SCD was 0.30 (95% CI 0.16–0.56; *I*^2^ = 0%; *P* = 0.0002) (Fig. 2). The DANISH trial also reported a significant reduction in SCD in the ICD group (HR 0.50; 95% CI 0.31–0.82; *P* = 0.005). However, this comparison was ICD and CRT‑D versus MT with or without CRT‑P.

### All-cause mortality, CRT-D versus medical therapy and CRT-D versus CRT-P

**Fig. 3:**
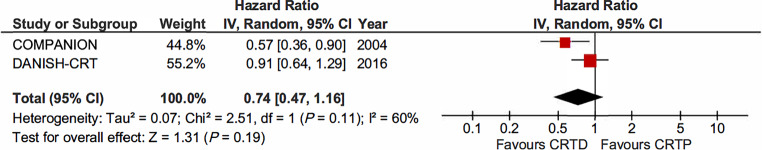
All-cause mortality among patients with non-ischaemic cardiomyopathy randomised to cardiac resynchronisation defibrillator (*CRT‑D*) versus cardiac resynchronisation pacemaker (*CRT‑P*). (*CI* confidence interval, *IV* interval variable, *random* random effect)

The COMPANION trial reported on the comparison of patients with CRT‑D versus those with MT (HR 0.50, 95% CI 0.29–0.88; *P* = 0.015). The DANISH trial reported on the comparison of patients with a CRT‑D versus those with a CRT‑P (HR 0.91, 95% CI 0.64–1.29; *P* = 0.59). Data on the comparison between CRT‑D and CRT‑P has recently been published by the COMPANION investigators (HR 0.54, 95% CI 0.34–0.86; *P* = 0.009). The pooled HR showed no reduction in all-cause mortality among patients treated with CRT‑D compared with those with CRT‑P (HR 0.74, 95% CI 0.47–1.16; *P* = 0.19) (Fig. 3).

### Estimates of absolute effects in the Dutch population

**Fig. 4:**
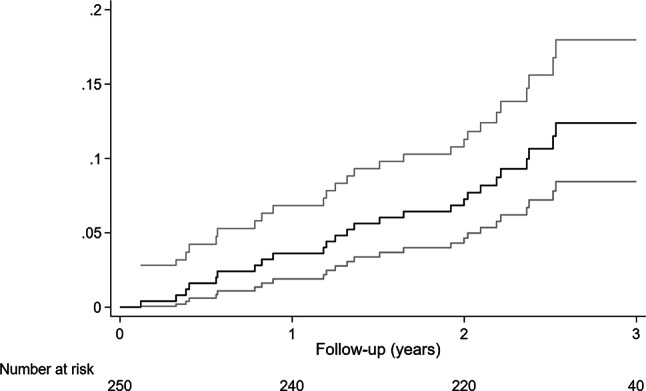
Cumulative mortality rate of patients with non-ischaemic cardiomyopathy and ICD-only therapy enrolled in the Dutch outcome in ICD therapy (*DO-IT*) study

Fig. 4 shows the cumulative mortality of NICMP patients with ICD-only therapy enrolled in the DO-IT registry, which was 3.6%, 7.3%, and 12.8% at 1, 2 and 3 years, respectively. Assuming that hazards of mortality were constant during each year, these observations correspond with hazards of mortality of 0.037, 0.039 and 0.062 in the three respective follow-up years. Based on the current meta-analysis, the hazards would have been 1/0.76 times higher (as the HR for the comparison of ICD-only versus MT was 0.76) if the DO-IT patients had been treated with MT only: 0.048, 0.052 and 0.081. Using these hazards, and applying the exponential survival model, had the DO-IT patients received MT, the expected cumulative mortality at 3 years is 16.5%. Hence, the estimated ARR comparing ICD-only versus MT after 3 years follow-up based on this Dutch registry is 3.7% and the NNT 27.0. In terms of life years gained (difference between the areas under the cumulative survival curves), the NNT is estimated at 19.3.

### Comparison with real-world clinical data

**Fig. 5:**
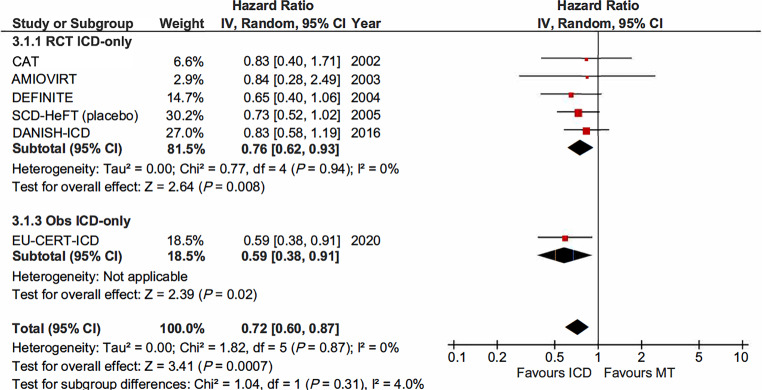
All-cause mortality among patients with non-ischaemic cardiomyopathy. Pooled data of the randomised clinical trials with prospective controlled study comparing implantable cardioverter-defibrillator (*ICD*) with medical therapy (*MT*). (*CI* confidence interval, *IV* interval variable, *random* random effect, *Obs* observational study, *RCT* randomised clinical trial)

The prospective, controlled study EU-CERT-ICD assessed the clinical effectiveness of primary prevention ICD therapy. Non-ischaemic cardiomyopathy was present in 35% of enrolled patients. All-cause mortality was significantly reduced in the ICD group compared with MT (HR 0.59, 95% CI 0.38–0.91; *P* = 0.017). When pooling data of the RCTs with EU-CERT-ICD, all-cause mortality was significantly reduced by use of an ICD (HR 0.72, 95% CI 0.60–0.87; *I*^2^ = 0%; *P* < 0.001) (Fig. 5).

**Fig. 6:**
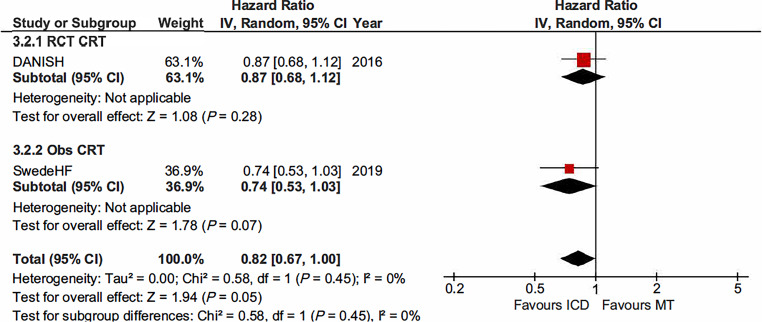
All-cause mortality among patients with non-ischaemic cardiomyopathy. Pooled data of the randomised clinical trials with prospective controlled study comparing implantable cardioverter-defibrillator (*ICD*) with medical therapy (*MT*) both with and without cardiac resynchronisation therapy (*CRT*). (*CI* confidence interval, *IV* interval variable, *random* random effect, *Obs* observational study, *RCT* randomised clinical trial)

The SwedeHF registry evaluated the association between primary prevention ICD therapy and all-cause mortality in a large, contemporary cohort of patients with HF and reduced LVEF. ICD recipients were propensity matched 1:1 to non-ICD recipients, both groups were with and without CRT. When pooling data of DANISH with SwedeHF, all-cause mortality was reduced in the ICD-group, including CRT, compared with MT (HR 0.82, 95% CI 0.67–1.00; I^2^; *P* = 0.05) (Fig. 6).

## Discussion

The current meta-analysis provides an important additional insight in ICD therapy for NICMP by stratifying according to CRT use. The major finding of our meta-analysis is that ICD-only therapy as primary prevention in patients with NICMP is associated with a 24% reduction in all-cause mortality and a 70% reduction in SCD compared with MT. In order to assess risk and benefit of ICD-only therapy in Dutch clinical practices, the pooled estimate was applied to the DO-IT registry. The 3‑year ARR is 3.7% (NNT 27.0) and in terms of life years gained (NNT 19.3). Considering CRT-eligible patients with NICMP, we found that there was no significant association with a reduction in all-cause mortality of CRT‑D versus CRT‑P.

### ICD-only therapy

In patients with NICMP not eligible for CRT, ICD-only therapy significantly reduces all-cause mortality (HR 0.76). Compared with the result of a previous meta-analysis prior to the DANISH trial (HR 0.74) [34], incorporation of ICD-only data from DANISH in the analysis only had a weak effect on survival benefit of ICD therapy. The EU-CERT-ICD study demonstrated that ICD-only therapy was associated with a 59% reduction in all-cause mortality in contemporary patients with NICMP [28]. When pooling the data of RCTs and the EU-CERT-ICD study, ICD-only therapy significantly reduced all-cause mortality by 28%. Based on these results, primary prophylactic ICD therapy should remain the standard therapy in patients with NICMP, LVEF ≤ 35%, and NYHA class II–III, and without an indication for CRT.

### Cardiac resynchronisation therapy

The COMPANION trial was the first study to demonstrate a significant reduction in all-cause mortality by CRT compared with MT in patients with NICMP and intraventricular conduction delay [33]. Only recently, the COMPANION investigators evaluated the outcomes of CRT‑D compared with CRT‑P by aetiology of HF. This post-hoc analysis found that in patients with NICMP, CRT‑D was associated with reduced all-cause mortality compared with CRT‑P [35]. In contrast, no difference in all-cause mortality between CRT‑D and CRT‑P was observed in the DANISH trial [6]. In pooled analysis of COMPANION and DANISH, CRT‑D was not significantly associated with a reduction in all-cause mortality in CRT-eligible patients with NICMP. Several aspects may contribute to the disparity in results among COMPANION and DANISH. Compared with DANISH, patients in COMPANION had more advanced HF; NYHA class III or IV and a mean baseline LVEF of 20%, while the majority of patients in DANISH had NYHA class II and a mean baseline LVEF of 25%. In addition, guideline-directed MT, including beta-blockers, angiotensin-converting enzyme inhibitors or angiotensin receptor blockers, and mineralocorticoid receptor antagonists, was more robust in DANISH. Newer medications such as sacubitril/valsartan and sodium-glucose cotransporter‑2 inhibitors have shown to reduce all-cause mortality [36, 37]. However, these drugs were not available for HF treatment in both DANISH and COMPANION and thus does not explain the difference in results between both trials. Important to note is that both trials were not specifically powered to assess whether the addition of ICD back-up would benefit CRT-eligible patients with NICMP.

The importance of ICD back-up in CRT patients has been evaluated in two meta-analyses. Barra et al. found a trend towards reduced all-cause mortality by CRT‑D in patients with NICMP (HR 0.79) [38]. The recent meta-analysis by Patel et al. found no significant reduction in all-cause mortality by CRT‑D compared with CRT‑P [39]. They found a pooled HR of 0.92 (95% CI 0.83–1.02) which was similar to the one observed in DANISH (HR 0.91, 95% CI 0.64–1.29). Despite the beneficial effects of CRT, mortality is not uniform among patients as CRT-candidates have heterogeneous risk profiles. Patients may have mild to severe HF, different HF aetiology, and different burden of various potentially co-existing comorbidities.

Previous studies have shown substantial risk of mortality in ICD patients who have concomitant non-cardiac comorbidities [40–42]. A meta-analysis of four RCTs evaluating the survival benefit of primary prevention ICDs demonstrated that patients with extensive comorbidity may experience less benefit from ICD compared with those with less comorbidity [43]. In the Cause of Death Analysis of Patients With Cardiac Resynchronization Therapy (CeRtiTuDe) registry, mortality was significantly higher among CRT‑P patients which was almost entirely attributed to non-SCD [44]. The CRT‑P patients were older, had more advanced HF and co-morbidities when compared with CRT‑D patients. In a post-hoc analysis of DANISH, ICD therapy was associated with reduced all-cause mortality in patients ≤ 70 years of age [45]. Older patients were more likely to die of non-sudden cardiac death.

### Impact on clinical practice

The evidence for mortality benefit by ICDs in patients with NICMP has always been less robust compared with patients with ischaemic cardiomyopathy. The results of DANISH suggested that ICDs may not reduce all-cause mortality and questioned even more the role of ICD therapy in NICMP. So which patients with NICMP might obtain a worthwhile benefit from prophylactic ICD therapy? Our meta-analysis provides important considerations of ICD therapy for this patient group. Considering patients who are eligible for CRT, the results of our analysis are concordant with DANISH; we found no significant reduction in all-cause mortality in CRT-eligible patients who received a CRT‑D compared with CRT‑P. For non-CRT-eligible patients with NICMP, ICD-therapy is associated with a significant reduction in all-cause mortality. We have to keep in mind that mortality risk is not uniform among patients. In addition, the absolute benefit of ICD therapy may have diminished with reductions in the absolute rate of cardiovascular death due to advances in MT and device therapy such as CRT.

At face value, patients who are older and who are afflicted by more comorbidities are less likely to benefit from ICD therapy either with or without CRT. In clinical practice, an individualised approach focusing on risk stratification may assist physicians in a shared decision-making process whether a patient will benefit from ICD therapy [26, 46, 47]. Of note, NICMP is a heterogeneous condition with a variety of causes, and the risk of life-threatening ventricular arrhythmias is higher in some conditions (e.g. sarcoidosis, phospholamban mutation). For patients with these specific conditions, models have been developed to assess the risk of SCD [48–50].

## Strengths and limitations

The strength of this meta-analysis is that the analysis on ICD benefit was stratified for CRT status and data were compared with real-world clinical data to perform a risk/benefit analysis. The primary limitation of this analysis is the absence of patient-level data, which limited the ability to assess ICD benefit in subgroups. The absence of patient-level data prevented the exploration of the impact of baseline mortality risk on ICD benefit. Considering CRT, studies reporting outcomes on CRT‑P versus CRT‑D were limited in number and sample size, which limits the ability to make conclusions in the CRT sub-group. In general, age and co-morbidities may confound whether ICD therapy with or without CRT may improve survival or not.

## Conclusion

The current meta-analysis supports the use of ICD for primary prevention of SCD in patients with NICMP who are not eligible for CRT. When applied to Dutch clinical practice, ICD-only therapy has a 3-year ARR of 3.7% (NNT 27.0) and in terms of life years gained (NNT 19.3). Considering CRT, we found no significant association with a reduction in all-cause mortality in patients with NICMP receiving CRT‑D as compared with CRT‑P. Further research is needed to assess the efficacy of CRT‑D in comparison to CRT‑P in patients with NICMP.

## Supplementary Information

Table providing the analytical characteristics and findings of previous published meta-analyses regarding the value of ICD therapy in non-ischaemic cardiomyopathy, and a flowchart showing the process of study selection

## References

[1] Al-Khatib SM, Stevenson WG, Ackerman MJ (2018). 2017 AHA/ACC/HRS guideline for management of patients with ventricular arrhythmias and the prevention of sudden cardiac death: a report of the American College of Cardiology/American Heart Association Task Force on Clinical Practice Guidelines and the Heart Rhythm Society. Heart Rhythm.

[2] Priori SG, Blomstrom-Lundqvist C, Mazzanti A (2015). 2015 ESC guidelines for the management of patients with ventricular arrhythmias and the prevention of sudden cardiac death: the Task Force for the Management of Patients with Ventricular Arrhythmias and the Prevention of Sudden Cardiac Death of the European Society of Cardiology (ESC)Endorsed by: Association for European Paediatric and Congenital Cardiology (AEPC). Europace.

[3] Bardy GH, Lee KL, Mark DB (2005). Amiodarone or an implantable cardioverter-defibrillator for congestive heart failure. N Engl J Med.

[4] Moss AJ, Zareba W, Hall WJ (2002). Prophylactic implantation of a defibrillator in patients with myocardial infarction and reduced ejection fraction. N Engl J Med.

[5] Desai AS, Fang JC, Maisel WH, Baughman KL (2004). Implantable defibrillators for the prevention of mortality in patients with nonischemic cardiomyopathy: a meta-analysis of randomized controlled trials. JAMA.

[6] Kober L, Thune JJ, Nielsen JC (2016). Defibrillator implantation in patients with nonischemic systolic heart failure. N Engl J Med.

[7] Akel T, Lafferty J (2017). Implantable cardioverter defibrillators for primary prevention in patients with nonischemic cardiomyopathy: a systematic review and meta-analysis. Cardiovasc Ther.

[8] Al-Khatib SM, Fonarow GC, Joglar JA (2017). Primary prevention implantable cardioverter defibrillators in patients with nonischemic cardiomyopathy: a meta-analysis. JAMA Cardiol.

[9] Barakat AF, Saad M, Elgendy AY (2017). Primary prevention implantable cardioverter defibrillator in patients with non-ischaemic cardiomyopathy: a&nbsp;meta-analysis of randomised controlled trials. BMJ Open.

[10] Cavalcanti R, Aboul-Hosn N, Morales G, Abdel-Latif A (2018). Implantable cardioverter defibrillator for the primary prevention of sudden cardiac death in patients with nonischemic cardiomyopathy. Angiology.

[11] Golwala H, Bajaj NS, Arora G, Arora P (2017). Implantable cardioverter-defibrillator for nonischemic cardiomyopathy: an updated meta-analysis. Circulation.

[12] Khan SU, Ghimire S, Talluri S (2018). Implantable cardioverter defibrillator in nonischemic cardiomyopathy: a systematic review and meta-analysis. J Arrhythm.

[13] Kolodziejczak M, Andreotti F, Kowalewski M (2017). Implantable cardioverter-defibrillators for primary prevention in patients with ischemic or nonischemic cardiomyopathy: a systematic review and meta-analysis. Ann Intern Med.

[14] Luni FK, Singh H, Khan AR (2017). Mortality effect of ICD in primary prevention of nonischemic cardiomyopathy: a meta-analysis of randomized controlled trials. J Cardiovasc Electrophysiol.

[15] Masri A, Hammadah M, Adelstein E, Jain S, Saba S (2017). Implantable cardioverter defibrillator in non-ischemic cardiomyopathy: a&nbsp;meta-analysis of randomized controlled trials. Cardiovasc Diagn Ther.

[16] Anantha Narayanan M, Vakil K, Reddy YN (2017). Efficacy of implantable cardioverter-defibrillator therapy in patients with nonischemic cardiomyopathy: a systematic review and meta-analysis of randomized controlled trials. JACC Clin Electrophysiol.

[17] Romero J, Chaudhary R, Garg J (2017). Role of implantable cardioverter defibrillator in non-ischemic cardiomyopathy: a&nbsp;systematic review and meta-analysis of prospective randomized clinical trials. J Interv Card Electrophysiol.

[18] Shun-Shin MJ, Zheng SL, Cole GD (2017). Implantable cardioverter defibrillators for primary prevention of death in left ventricular dysfunction with and without ischaemic heart disease: a&nbsp;meta-analysis of 8567&nbsp;patients in the 11 trials. Eur Heart J.

[19] Stavrakis S, Asad Z, Reynolds D (2017). Implantable cardioverter defibrillators for primary prevention of mortality in patients with nonischemic cardiomyopathy: a meta-analysis of randomized controlled trials. J Cardiovasc Electrophysiol.

[20] Wolff G, Lin Y, Karathanos A (2017). Implantable cardioverter/defibrillators for primary prevention in dilated cardiomyopathy post-DANISH: an updated meta-analysis and systematic review of randomized controlled trials. Clin Res Cardiol.

[21] Alba AC, Foroutan F, Duero Posada J (2018). Implantable cardiac defibrillator and mortality in non-ischaemic cardiomyopathy: an updated meta-analysis. Heart.

[22] Beggs SAS, Jhund PS, Jackson CE, McMurray JJV, Gardner RS (2018). Non-ischaemic cardiomyopathy, sudden death and implantable defibrillators: a&nbsp;review and meta-analysis. Heart.

[23] Romero J, Diaz JC, Grushko M (2018). Clinical impact of implantable cardioverter-defibrillator in primary prevention of total mortality in non-ischaemic cardiomyopathy: results from a&nbsp;meta-analysis of prospective randomized clinical trials. Europace.

[24] Siddiqui WJ, Aggarwal S, Rafique M (2018). Prophylactic use of the implantable cardioverter-defibrillator and its effect on the long-term survival, cardiovascular and sudden cardiac death in nonischemic cardiomyopathy patients—a systematic review and meta-analysis. Heart Fail Rev.

[25] DerSimonian R, Laird N (1986). Meta-analysis in clinical trials. Control Clin Trials.

[26] Verstraelen TE, van Barreveld M, van Dessel PHFM (2021). Development and external validation of prediction models to predict implantable cardioverter-defibrillator efficacy in primary prevention of sudden cardiac death. Europace.

[27] Pezawas T, Grimm M, Ristl R (2015). Primary preventive cardioverter-defibrillator implantation (Pro-ICD) in patients awaiting heart transplantation. A prospective, randomized, controlled 12-year follow-up study. Transpl Int.

[28] Zabel M, Willems R, Lubinski A (2020). Clinical effectiveness of primary prevention implantable cardioverter-defibrillators: results of the EU-CERT-ICD controlled multicentre cohort study. Eur Heart J.

[29] Schrage B, Uijl A, Benson L (2019). Association between use of primary-prevention implantable cardioverter-defibrillators and mortality in patients with heart failure: a prospective propensity score-matched analysis from the Swedish Heart Failure Registry. Circulation.

[30] Bansch D, Antz M, Boczor S (2002). Primary prevention of sudden cardiac death in idiopathic dilated cardiomyopathy: the Cardiomyopathy Trial (CAT). Circulation.

[31] Strickberger SA, Hummel JD, Bartlett TG (2003). Amiodarone versus implantable cardioverter-defibrillator: randomized trial in patients with nonischemic dilated cardiomyopathy and asymptomatic nonsustained ventricular tachycardia—AMIOVIRT. J Am Coll Cardiol.

[32] Kadish A, Dyer A, Daubert JP (2004). Prophylactic defibrillator implantation in patients with nonischemic dilated cardiomyopathy. N Engl J Med.

[33] Bristow MR, Saxon LA, Boehmer J (2004). Cardiac-resynchronization therapy with or without an implantable defibrillator in advanced chronic heart failure. N Engl J Med.

[34] Theuns DA, Smith T, Hunink MG, Bardy GH, Jordaens L (2010). Effectiveness of prophylactic implantation of cardioverter-defibrillators without cardiac resynchronization therapy in patients with ischaemic or non-ischaemic heart disease: a&nbsp;systematic review and meta-analysis. Europace.

[35] Doran B, Mei C, Varosy PD (2021). The addition of a defibrillator to resynchronization therapy decreases mortality in patients with nonischemic cardiomyopathy. JACC Heart Fail.

[36] McMurray JJ, Packer M, Desai AS (2014). Angiotensin-neprilysin inhibition versus enalapril in heart failure. N Engl J Med.

[37] Zannad F, McMurray JJ, Krum H (2011). Eplerenone in patients with systolic heart failure and mild symptoms. N Engl J Med.

[38] Barra S, Providencia R, Tang A (2015). Importance of implantable cardioverter-defibrillator back-up in cardiac resynchronization therapy recipients: a systematic review and meta-analysis. J Am Heart Assoc.

[39] Patel D, Kumar A, Black-Maier E (2021). Cardiac resynchronization therapy with or without defibrillation in patients with nonischemic cardiomyopathy: a systematic review and meta-analysis. Circ Arrhythm Electrophysiol.

[40] Theuns DA, Schaer BA, Soliman OI (2011). The prognosis of implantable defibrillator patients treated with cardiac resynchronization therapy: comorbidity burden as predictor of mortality. Europace.

[41] Boriani G, Berti E, Belotti LM (2016). Cardiac device therapy in patients with left ventricular dysfunction and heart failure: ‘real-world’ data on long-term outcomes (mortality, hospitalizations, days alive and out of hospital). Eur J Heart Fail.

[42] Ruwald AC, Vinther M, Gislason GH (2017). The impact of co-morbidity burden on appropriate implantable cardioverter defibrillator therapy and all-cause mortality: insight from Danish nationwide clinical registers. Eur J Heart Fail.

[43] Steinberg BA, Al-Khatib SM, Edwards R (2014). Outcomes of implantable cardioverter-defibrillator use in patients with comorbidities: results from a&nbsp;combined analysis of 4 randomized clinical trials. JACC Heart Fail.

[44] Marijon E, Leclercq C, Narayanan K (2015). Causes-of-death analysis of patients with cardiac resynchronization therapy: an analysis of the CeRtiTuDe cohort study. Eur Heart J.

[45] Elming MB, Nielsen JC, Haarbo J (2017). Age and outcomes of primary prevention implantable cardioverter-defibrillators in patients with nonischemic systolic heart failure. Circulation.

[46] Bilchick KC, Wang Y, Curtis JP (2020). Modeling defibrillation benefit for survival among cardiac resynchronization therapy defibrillator recipients. Am Heart J.

[47] Theuns DAMJ, Schaer BA, Caliskan K (2021). Application of the heart failure meta-score to predict prognosis in patients with cardiac resynchronization defibrillators. Int J Cardiol.

[48] Cadrin-Tourigny J, Bosman LP, Nozza A (2019). A new prediction model for ventricular arrhythmias in arrhythmogenic right ventricular cardiomyopathy. Eur Heart J.

[49] O’Mahony C, Jichi F, Pavlou M (2014). A novel clinical risk prediction model for sudden cardiac death in hypertrophic cardiomyopathy (HCM risk-SCD). Eur Heart J.

[50] Verstraelen TE, van Lint FHM, Bosman LP (2021). Prediction of ventricular arrhythmia in phospholamban p.Arg14del mutation carriers-reaching the frontiers of individual risk prediction. Eur Heart J.

