# Supplementary material for: Implantable defibrillator therapy and mortality in patients with non-ischaemic dilated cardiomyopathy: An updated meta-analysis and effect on Dutch clinical practice by the Task Force of the Dutch Society of Cardiology
Source: Neth Heart J. 2022 Sep 6;31(3):89–99. doi: 10.1007/s12471-022-01718-3 (PMC9950314; doi:10.1007/s12471-022-01718-3)

**Supplementary Table S1** Characteristics of meta-analyses post DANISH trial regarding value of ICD therapy in non-ischaemic cardiomyopathy.

| **Author** | **Year** | **Studies**  **(n)** | **Participants**  **(n)** | **Comparison** | **Result**  **all-cause mortality** | **Remark** |
| --- | --- | --- | --- | --- | --- | --- |
| Akel *et al.* (33) | 2017 | 5 | 2,573 | MT vs. ICD +CRTD | HR 0.80 (0.67, 0.96) § | Exclusion of COMPANION; data of DANISH pooled; SCD-HeFT analysis (ICD vs. placebo) |
| Al-Khatib *et al.*(34) | 2017 | 4 | 1,874 | MT vs. ICD-only | HR 0.75 (0.61, 0.93) §‡ | Exclusion of AMIOVIRT; SCD-HeFT analysis incorrect number of ICD and OMT cohort |
| Barakat *et al*.(35) | 2017 | 5 | 2,573 | MT vs. ICD + CRTD | HR 0.79 (0.64, 0.93) ‡ | Exclusion of COMPANION; data of DANISH pooled; SCD-HeFT analysis (ICD vs. placebo) |
| Cavalcanti *et al.*(36) | 2017 | 5 | 1,928 | MT vs. ICD-only | RR 0.79 (0.66, 0.95) § | SCD-HeFT analysis (ICD vs. placebo) |
| Golwala *et al.*(37) | 2017 | 6 | 2,970 | MT vs. ICD + CRTD  MT vs. ICD-only  CRT | HR 0.77 (0.64, 0.91) ‡  HR 0.76 (0.62, 0.94) ‡  HR 0.70 (0.39, 1.26) ‡ | SCD-HeFT analysis (ICD vs. placebo); CRT sub-analysis COMPANION (CRTD vs. OMT) and DANISH (CRTD vs. CRTP) |
| Khan *et al.*(38) | 2017 | 6 | 3,388 | MT vs. ICD + CRTD | RR 0.74 (0.56, 0.97) §‡ | Data of DANISH pooled; SCD-HeFT analysis (ICD vs. placebo); CRT sub-analysis COMPANION (CRTD vs. OMT) and DANISH (CRTD vs. CRTP); HR’s entered for analysis |
| Kolodziejczak *et al.*(39) | 2017 | 5 | 2,992 | MT vs. ICD + CRTD | HR 0.81 (0.72, 0.91) ‡ | Exclusion of COMPANION; data of DANISH pooled; SCD-HeFT analysis (ICD vs. placebo + amiodarone), incorrect number of deaths |
| Luni *et al.*(40) | 2017 | 6 | NR | MT vs. ICD + CRTD | OR 0.76 (0.64, 0.91) § | Data of DANISH pooled; COMPANION analysis (CRTD vs. CRTP); SCD-HeFT analysis (ICD vs. placebo); HR’s entered for analysis |
| Masri *et al.*(41) | 2017 | 6 | 2,867 | MT vs. ICD + CRTD | RR 0.76 (0.66, 0.91) ‡ | Data of DANISH pooled; COMPANION analysis (CRTD vs. CRTP), incorrect number CRTP; SCD-HeFT analysis (ICD vs. placebo); HR’s entered for analysis |
| Narayanan *et al.*(42) | 2017 | 6 | 3,544 | MT vs. ICD-only  CRTP vs. CRTD | RR 0.76 (0.63, 0.91) §‡  RR 0.74 (0.47, 1.16) §‡ | SCD-HeFT analysis (ICD vs. placebo + amiodarone); CRT analysis (CRTP vs. CRTD), COMPANION incorrect number CRT cohorts |
| Romero *et al.*(43) | 2017 | 5 | 2,573 | MT vs. ICD + CRTD | RR 0.84 (0,71, 0.99) § | Exclusion of COMPANION; data of DANISH pooled; SCD-HeFT analysis (ICD vs. placebo) |
| Shun-Shin *et al.*(44) | 2017 | 6 * | 3,128 * | MT vs. ICD + CRTD | HR 0.76 (0.64, 0.90) ‡ | Data of DANISH pooled; COMPANION analysis (CRTD vs. OMT) incorrect number MT cohort; SCD-HeFT analysis (ICD vs. placebo) |
| Stavrakis *et al.*(45) | 2017 | 6 | 2,967 | MT vs. ICD + CRTD | HR 0.78 (0.66, 0.92) ‡ | Data of DANISH pooled; COMPANION analysis (CRTD vs. CRTP); SCD-HeFT analysis (ICD vs. placebo) |
| Wolff *et al.*(46) | 2017 | 5 | 2,992 | MT vs. ICD + CRTD | OR 0.77 (0.64, 0.93) § | Exclusion of COMPANION; data of DANISH pooled; SCD-HeFT analysis (ICD vs. placebo + amiodarone) |
| Alba *et al.*(47) | 2018 | 6 | 3,011 | MT vs. ICD + CRTD | RR 0.84 (0.73, 0.96) §  HR 0.78 (0.66, 0.92) § | Exclusion of COMPANION; data of DANISH pooled; inclusion of Pro-ICD (n=19); SCD-HeFT analysis ICD vs. placebo + ICD vs. amiodarone estimated from Kaplan-Meier analysis and ICD cohort entered twice |
| Beggs *et al.*(48) | 2018 | 6 | 2,970 | MT vs. ICD + CRTD | RR 0.76 (0.65, 0.91) § | Data of DANISH pooled; SCD-HeFT analysis (ICD vs. placebo); HR’s entered for analysis |
| Romero *et al.*(49) | 2018 | 6 | 3,128 | MT vs. ICD + CRTD | RR 0.79 (0.68, 0.92) ‡ | Data of DANISH pooled; COMPANION analysis (CRTD vs. CRTP); SCD-HeFT analysis (ICD vs. placebo) |
| Siddiqui *et al.*(50) | 2018 | 6 | 3,389 | MT vs. ICD + CRTD | OR 0.74 (0.62, 0.80) ‡ | Data of DANISH pooled; COMPANION analysis (CRTD vs. OMT); SCD-HeFT analysis (ICD vs. placebo + amiodarone) incorrect number of deaths |
| Effect measures: *§* fixed effects, *‡* random effects  * Meta-analysis consisted of 11 trials on primary and secondary prevention in patients with ischaemic and non-ischaemic heart disease.  *HR* hazard ratio, *OR* odds ratio, *RR* risk ratio, *CRTD* cardiac resynchronisation defibrillator, *CRTP* cardiac resynchronisation pacemaker, *ICD* implantable cardioverter-defibrillator, *MT* medical therapy, *NR* not reported | | | | | | |

**Supplemetary Fig. S1** Process of study selection


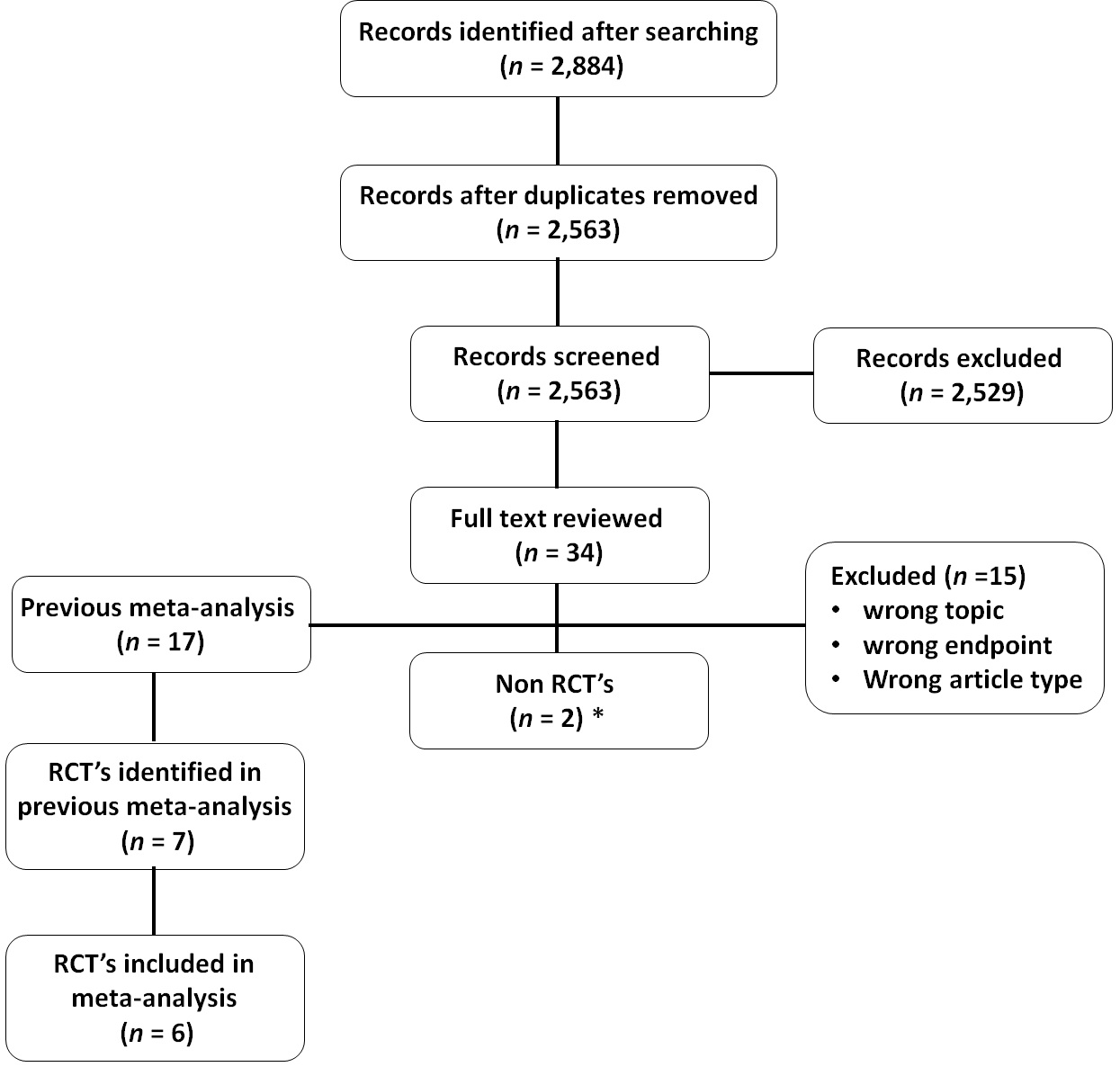

Supplement: Supplementary file 1 — Table providing the analytical characteristics and findings of previous published meta-analyses regarding the value of ICD therapy in non-ischaemic cardiomyopathy, and a flowchart showing the process of study selection [file 12471_2022_1718_MOESM1_ESM.docx]
